# Individual methanogenic granules are whole-ecosystem replicates with reproducible responses to environmental cues

**DOI:** 10.1186/s40793-024-00615-z

**Published:** 2024-09-09

**Authors:** Anna Trego, Sarah O’Sullivan, Vincent O’Flaherty, Gavin Collins, Umer Zeeshan Ijaz

**Affiliations:** 1 Sustainable World Section, School of Biological and Chemical Sciences, University of Galway, University Road, Galway, H91 TK33 Ireland; 2 Water Engineering Group, School of Engineering, The University of Glasgow, Oakfield Avenue, Glasgow, G12 8LT UK

**Keywords:** Anaerobic digestion, Biofilms, Community assembly, Microbial communities, Sludge granules, Wastewater

## Abstract

**Background:**

In this study, individual methanogenic (anaerobic), granular biofilms were used as true community replicates to assess whole-microbial-community responses to environmental cues. The aggregates were sourced from a lab-scale, engineered, biological wastewater treatment system, were size-separated, and the largest granules were individually subjected to controlled environmental cues in micro-batch reactors (μBRs).

**Results:**

Individual granules were identical with respect to the structure of the active community based on cDNA analysis. Additionally, it was observed that the active microbial community of individual granules, at the depth of 16S rRNA gene sequencing, produced reproducible responses to environmental changes in pH, temperature, substrate, and trace-metal supplementation. We identified resilient and susceptible taxa associated with each environmental condition tested, as well as selected specialists, whose niche preferences span the entire trophic chain required for the complete anaerobic degradation of organic matter.

**Conclusions:**

We found that single anaerobic granules can be considered highly-replicated whole-ecosystems with potential usefulness for the field of microbial ecology. Additionally, they act as the smallest whole-community unit within the meta-community of an engineered bioreactor. When subjected to various environmental cues, anaerobic granules responded reproducibly allowing for rare or unique opportunities for high-throughput studies testing whole-community responses to a wide range of environmental conditions.

**Supplementary Information:**

The online version contains supplementary material available at 10.1186/s40793-024-00615-z.

## Background

Microbial communities underpin biogeochemical cycles of Earth’s soil, oceans, and atmosphere, providing ecosystem services and functions for plants, animals, and humans. However, our understanding of their structure–function relationships and the mechanisms by which they assemble remains highly limited. Recent studies have refined increasingly innovative approaches to study spatio-temporal phylogenetic and functional dynamics of environmental microbiomes [1]. Moreover, the continued development of null models for the identification and quantification of community assembly mechanisms has helped elucidate the ongoing processes shaping microbiome structure and function [2–5]. An ongoing challenge however, will be to determine, and model, the response of microbial communities to environmental change, including changing climates.

Biotechnological applications, in which mixed-species consortia are applied for specific functions, may provide useful model systems, having clear functional parameters and offering controlled environments [6]. An important feature of many microbial communities is aggregation, not only into biofilm slimes but also into structured ‘flocs’ and highly-organized granules. Several important examples are seen across the natural and built environment, including methanogenic, aerobic, anammox, phototrophic and hydrogenic granular biofilms in wastewater treatment technologies [7, 8], as well as the pink berry [9] and ‘lake ball’ [10] granules observed in natural systems. Such aggregates are robust, and technologically advantageous over planktonic communities, due to: enhanced retention of constituent cells; protection of some organisms against local environmental changes; and the enmeshment of species supporting mutualistic and syntrophic relationships underpinning otherwise thermodynamically impossible reactions [11]. In those respects, architecturally organized granules, and the granulation phenomenon [12], support in fascinating ways the types of interdependent microbial relationships that underpin a variety of modern environmental biotechnologies.

Methanogenic granules, in particular, harbor consortia of multiple microbial trophic groups collectively pushing, and funneling, carbon through successive pathways toward production of biogas or other high-value intermediates [13, 14], facilitating wastewater treatment and biorefinery innovations. This is specifically enabled by the highly-organized spatial distribution of trophic groups in methanogenic granules [15]. In the same way that soil aggregates have been considered whole (i.e. complete) communities, and important for understanding ‘fine-scale’ community structure [16], granular aggregates may be equally instructive regarding community ecology in anaerobic ecosystems. Moreover, the granules are the smallest representative unit of the whole microbial community, potentially providing an elegant solution to the challenges of whole-community sampling and analysis [17].

For those reasons, interest in the ecology of granules and other aggregates is expanding beyond applications in engineered systems. Recently, Leventhal et al*.* [17] used aerobic granules as replicated communities to investigate the extent to which intrinsic or extrinsic factors influence microbiome structure – and at which degree of taxonomic resolution community types can be resolved. Clear community types were not observed at the genus-level, but were apparent at strain-level, indicating strain-level diversification [17]. It will be useful to further characterize single granules through an ecological lens. This presents opportunities to leverage granular biofilms as whole microbial communities, in a range of compelling ways to address pressing questions on the structure–function and assembly of complex microbiomes.

In this study we investigated whether individual methanogenic granules were, indeed, discreet, and complex, whole microbial communities. Furthermore, we examined the impact of specific stress disturbances on individual aggregates, thereby assessing the utility of single granules for ‘high-throughput’ studies of microbial ecology. Specifically, individual granules were subjected to a range of environmental pressures in a micro-batch reactor (μBR) incubations. Changes to the active microbiomes were then investigated using cDNA from the incubations. Individual granules were, indeed, whole microbial communities, and can even be considered ‘patch’ communities within the meta-community of a full-scale, methanogenic bioreactor. Moreover, the experiments offer an example of how whole microbial community replicates may be comprehensively monitored.

## Results

### Within-group consistency of individual granule replicates

**Fig. 1:**
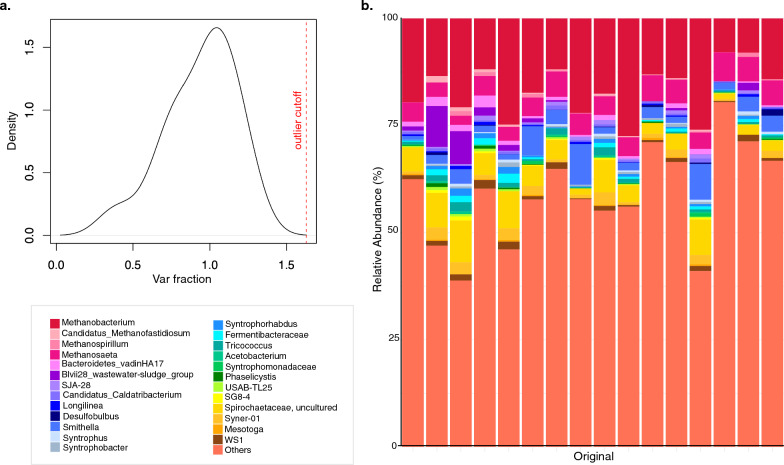
Similarity and structure of single-granule aggregates. The density plot (**a**) was generated using the codaSeq.Outlier() function from https://github.com/ggloor/CoDaSeq, which after a CLR (Centralized Log Transform) of the samples (*n* = 16 original granules), returned a list of proportional contribution to group variance, with outliers defined as those contributing greater than the median plus twice the interquartile range of the sample variance to the total (> 2 IQR away from the median). The density plot shows this cutoff as the dotted line in the density plot. The relative abundances (**b**) of the 25 most abundant genera in the active fraction of the microbiome of the 16 replicates are shown in a stacked bar plot, where ‘others’ refers to all others not included in the ‘top-25’

Sequencing the cDNA generated from the RNA of the 16 individual aggregates—henceforth referred to as ‘original—allowed us to determine whether individual granules represent highly replicated microbiomes. To determine how similar these 16 replicates were, we tested for within-group consistency. This was done by determining how much each sample contributed to the group’s total variance, with outliers found outside the distribution. No outliers (no samples to the right of the outlier cutoff) were identified, indicating all 16 individual aggregates were highly similar (Fig. 1).

**Fig. 2:**
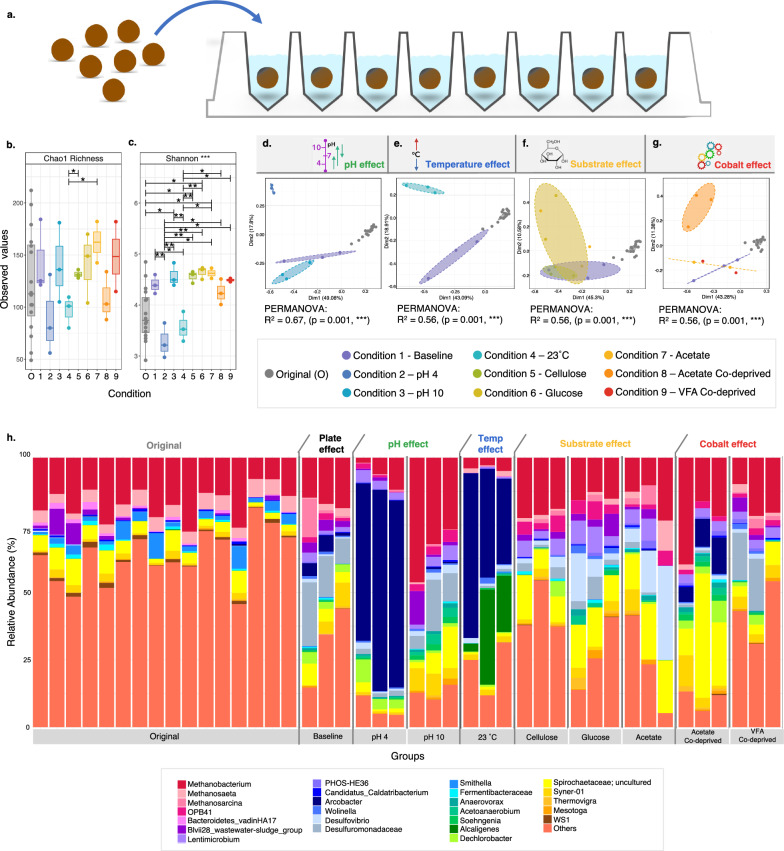
Active (RNA-based) microbial diversity and community structure according to variances in the 16S rRNA gene from single-granule, whole-ecosystems from untreated, original (*n* = 16) granules, as well as granules (*n* = 3) treated under various, pH, substrate, temperature and cobalt-deprivation conditions in the mBRs (**a**). *Alpha diversity*: where (**b**) is a box plot of the rarefied Chao1 richness and (**c**) the Shannon Entropy; *Beta diversity*: PCoA plots for changes according to (**d**) pH, (**e**) temperature, (**f**) substrates and (**g**) cobalt depravation using Bray–Curtis distances; community structure based on (**h**) the relative abundance of the top-25 most abundant genera from across each category, where ‘others’ refers to all others not included in the ‘top-25’; lines for figures (**b**,**c**) connect two categories where the differences were significant (ANOVA) with * (p < 0.05), ** (p < 0.01), or *** (p < 0.001). Ellipses for (**d**–**g**) are drawn at a 95% CI

Moreover, they clustered tightly when plotted on PCoA (Fig. 2), with an almost imperceptible confidence interval (CI) ellipse. The active community structure of these 16 replicates was comprised of abundant methanogenic archaea belonging to the phyla *Euryarchaeota* and *Haloarchaeota* (Fig. 1). Indeed, about 25% of the active community structure comprised of *Methanobacterium*, ‘*Candidatus* methanofastidiosum’, *Methanospirillum* and *Methanosaeta (Methanothrix)*. While there were slight variations in the relative abundances of these taxa the overall community structure was highly replicable.

### Response of whole microbial communities to specific environmental cues

**Fig. 3:**
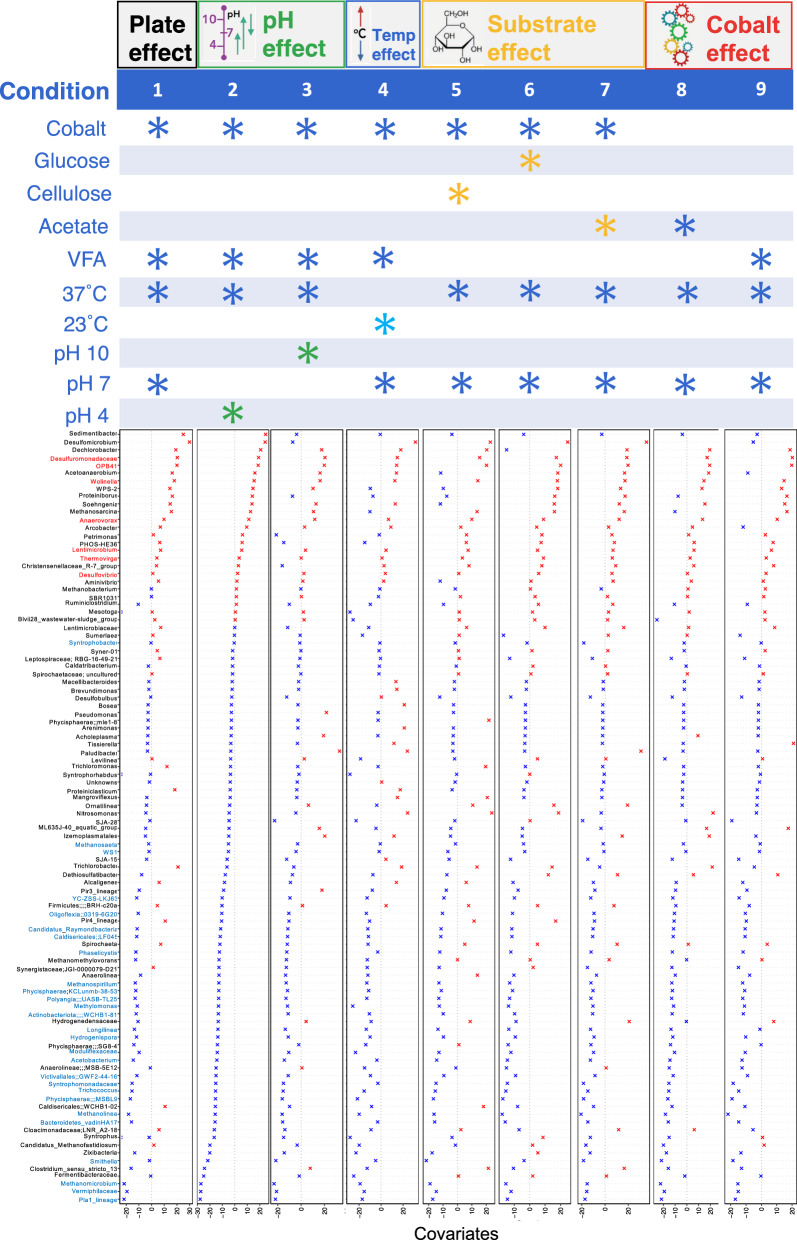
Susceptible and resilient taxa. The top chart details the specifics of the nine environmental conditions tested by the μBR experiments. Standard conditions are indicated by dark blue stars, while colored stars represent the changing variable tested by that condition. Underneath, are the ß-coefficients returned from GLLVM when applied to the top-100 most abundant genera and against each of the nine environmental conditions. ß-coefficients which are positively associated with the microbial abundance of a particular genus are colored red while negative associations are colored blue. Additionally, those genera whose names are highlighted in red were found positive for every condition (resilient), while those highlighted in blue were negative for every condition (susceptible). Since collation of ASVs was performed at the genus level, all those ASVs that cannot be categorized based on taxonomy are collated under “unknowns”

Single granules were then separately incubated in μBRs under select environmental cues (Fig. 3). Five different ‘effects’ were tested by applying nine distinct environmental conditions. Firstly, the ‘plate effect’ was examined by applying the same lab-scale bioreactor source conditions to the μBR granules (Condition 1). This provided a set of baseline conditions, which could be used to compare with the other eight conditions. When compared to the original granules, Condition 1 showed the effect that individual plate incubation induced on the microbial community. Next, the ‘pH effect’ altered the baseline conditions by adjusting the pH to either pH 4 (Condition 2) or pH 10 (Condition 3). The ‘temperature effect’ was examined by changing the baseline conditions to 23ºC (Condition 4). The ‘substrate effect’ tested three alternate substrates: cellulose, glucose and acetate (Conditions 5, 6 & 7), and, finally, the ‘cobalt effect’ tested for changes that occur when the incubations were deprived of cobalt, a vital trace element for anaerobic digestion (Conditions 8 & 9).

Chao1 richness revealed only very minor fluctuations in rarefied richness between any of the groups. Significant differences were, however, observed in terms of the Shannon entropy. In particular, changes in pH and temperature induced a significant shift in alpha diversity (Fig. 2). Beta diversity analysis showed significant clustering of samples based on the environmental condition applied (PERMANOVA: p = 0.001, ***). This indicated that the responses to these environmental cues were replicable at the single-granule level. Significant changes were observed between the original granules, and the baseline (Condition 1; plate effect) conditions. Temperature and pH changes resulted in distinct clusters, while changes in substrate and cobalt resulted in some cluster overlap. Changing the substrate from a volatile fatty acid (VFA) mix (Condition 1) to cellulose (Condition 5) resulted in minimal changes to the beta diversity. However, changing the substrate to glucose (Condition 6) or acetate (Condition 7) resulted in a more pronounced shift in community diversity. Regarding cobalt addition/deprivation, when the granules were supplied with VFA mix as a substrate (Condition 1 vs. Condition 9), cobalt deprivation had a minimal effect on the beta diversity. However, when the granules were supplied with acetate as a substrate (Condition 7 vs. Condition 8), cobalt deprivation significantly changed the community diversity.

Changed diversity was also reflected in the relative abundances of the 25 most abundant genera (Fig. 3). The relatively most abundant taxa across all samples all were methanogenic archaea: *Methanobacterium*, *Methanosaeta*, and *Methanosarcina*. However, the various environmental conditions we applied stimulated pronounced community shifts. Shifting from a lab-scale reactor system to the individual μBR configuration resulted in elevated relative abundances of several groups, including *Desulfuromonadaceae* and *Dechlorobacter*. Notably, low pH (Condition 2), the lower temperature (Condition 4), and acetate-supplied, cobalt-deprived (Condition 8) incubations increased the relative abundance of *Arcobacter*. Lower pH and temperatures also resulted in lower relative abundances of the methanogenic archaea. *Alcaligens*, which was rare in all samples, was relatively more abundant at the lower temperature.

Additionally, VFA profiles were monitored during these incubations as a means of targeted metabolomics. VFA degradation capacity appeared most complete in incubations at pH 7 (Fig. S1). Residual VFA were detected in incubations at pH 4 and 10. Lowering the temperature resulted in lower rates of VFA consumption (Fig. S1). At 37ºC, 91% of the acetic acid was consume, whilst at 23ºC total acetic acid consumption was 63%. Cobalt-deprivation resulted in lower rates of VFA utilization (Fig. S1) compared to cobalt-supplied conditions. The most pronounced effect was on acetic acid consumption, which was significantly (p = 0.043) lower with cobalt deprivation. Acetate-supplied granules produced a significantly different (p = 0.008) acetic acid profile to granules supplied with the more generalized VFA mixture. Furthermore, glucose-supplied granules had significantly (p = 0.002) increased fermentation rates than did granules supplied with cellulose (Fig. S2).

### Resilient and susceptible groups

To determine which taxa were significantly changing under the nine conditions tested, we applied a linear regression model GLLVM (generalized linear latent variable model) against the 100 most abundant taxa. The ‘original’ granules were used as a reference for change. All of the 100 most abundant taxa showed changes in response to the conditions (Fig. 3). For most of these taxa, some conditions induced a positive response, whilst others induced a negative response. Notably, most of the responses appeared to be negative. A number of taxa, however, were identified as either *resilient*, having positive responses to all conditions, or *susceptible*, having negative responses to all conditions. The resilient taxa were: *Desulfuromonadaceae*, *OPB41*, *Wolinella*, *Anaerovorax*, *Lentimicrobium*, *Thermovigra* and *Desulfovibrio*. There were more susceptible taxa, but several notable ones included: *Syntrophobacter*, *Methanosaeta*, *Longilinea*, *Acetobacterium*, *Syntrophomonadaceae*, *Trichococcus*, *Methanolinea*, *Bacteroidetes*_*vadinHA17*, *Smithella* and *Methanomicrobium*.

### Stable community subset and core fractions of the microbiome

A common way to evaluate a microbial community is to consider the members making up the core, or stable fraction. Core analysis, in particular, is very popular, and there are several ways of identifying a core community [18, 19]. Here we have applied two methods: (i) a new tool called ensemble quotient optimization (EQO) which identified a ‘stable’ subset of the microbiome wherein the abundances of individual taxa may fluctuate, but the overall abundance of the subset is preserved across the experimental conditions applied [20]; and (ii) a condition-specific occupancy model [21] to identify members of the core microbiome and assembly mechanisms of core-ASVs.

**Fig. 4:**
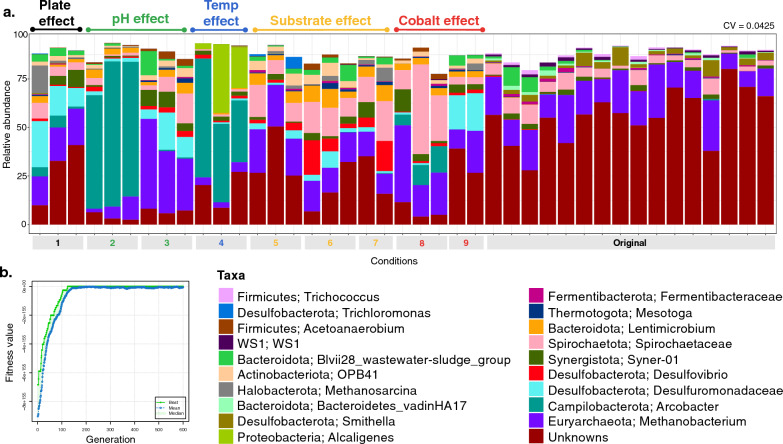
Stable ensemble of the active microbiome of all samples returned by the EQO algorithm in uniform phenotypic variable mode with (**a**) showing the relative abundance profiles (coefficient of variation (CV) values given at the top-right), and (**b**) showing the fitness value evolution of the genetic algorithm in finding these ensembles, highlighting the convergence to a steady state solution

The EQO algorithm was set to return 20 genera which can be considered stable community members (Fig. 4). Despite the perturbations applied experimentally, these members compensate, or equilibrate the system by preserving the overall abundance of the subset. This subset accounted for ~ 80–90% of the total relative abundance of the community. Most of these genera were also identified as members of the top-25 most abundant taxa, with major contributors being *Methanobacterium*, *Arcobacter*, *Desulfuromonadaceae* and *Spirochaetaceae*. The nine environmental conditions induced specific changes in the relative abundances of these taxa. For example, as noted previously, Condition 2 (pH 4), Condition 4 (temperature 23ºC) and Condition 8 (acetate-fed cobalt deprivation) all resulted in an increased relative abundance of *Arcobacter*, which was not observed to the same extent under any other condition. Similarly, the lower temperature (Condition 4) resulted in relatively more *Alcaligenes*—which was observed only under this condition.

**Fig. 5:**
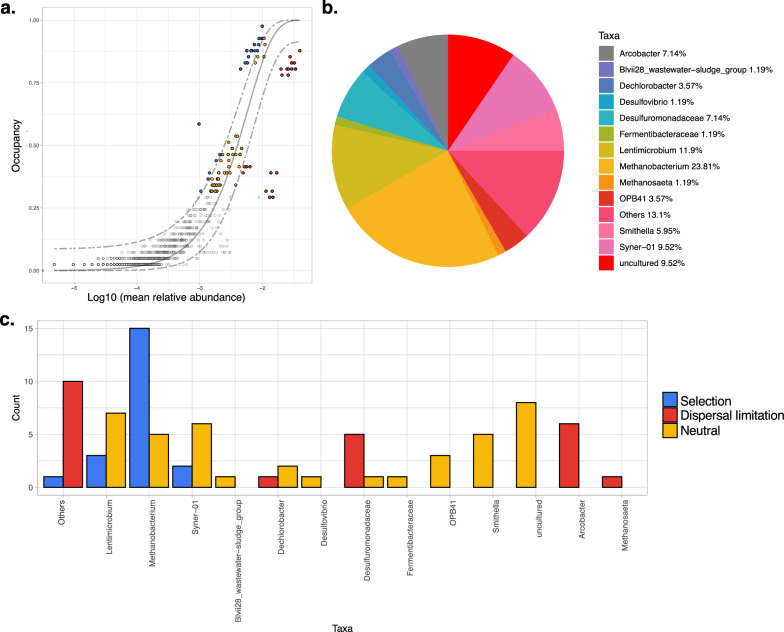
Identification and assembly of the core microbiome. The core was identified using (**a**) species occupancy abundance diagrams. These incorporated (for each temperature) a time-specific occupancy model (time being the four time-points sampled during Phase 1). Once ASV rankings were obtained based on ASV occupancy within these compartments as well as their replicate consistency, Bray–Curtis similarity was calculated for the whole dataset, and also for only the top-ranked taxa. The contribution of the top-ranked taxa was divided by the total Bray–Curtis similarity to calculate a percent contribution of the prospective core set to beta diversity. The next ranked taxon was added consecutively to find the point in the ranking at which adding one more taxon offered diminishing returns on explanatory value for beta diversity. The blue dotted line represents the “last 2% decrease” criteria where ASVs are incorporated in the core subset until there is no more than 2% decrease in beta diversity. Independently, a neutral model was fitted and those ASVs that fall within the 95% confidence interval were colored yellow. Non-neutral ASVs that fall above the model (blue) were selected by the host environment, while those below the neutral model (red) were assembled via dispersal limitation. Pie charts in (**b**) represent the genus-level assignments of the core ASVs. Finally, (**c**) the bar plot counts the number of neutral/non-neutral ASVs at genus-level

Core microbiome analysis returned 12 genera containing ASVs identified as core (Fig. 5). *Methanobacterium* made up the largest fraction of the core (23.81%), followed by *Lentimicrobium* (11.9%) and *Syner*-*01* (9.52%). When a neutral model for microbial community assembly was fit against the core ASVs, a majority of these ASVs (spanning several genera) were found to be assembled neutrally. However, the most abundant groups (*Methanobacterium*, *Lentimicrobium* and *Syner-01*) included ASVs assembled via selection processes, whilst genera such as *Arcobacter* and *Methanosaeta* contained core ASVs only assembled via dispersal limitation.

### Identification of specialist groups

The concept of niche has been a foundational idea in microbial ecology. It attempts to explain how environmental conditions and species interactions regulate the abundance and/or activity of a species. Niche breadth (B_N_) analysis was applied to identify generalists (B_N_ = 1), which have a broad, non-discriminatory niche, and specialists (B_N_ approaches 1/R; where R is the number of available resource states), which have a narrow, discriminatory niche.

**Fig. 6:**
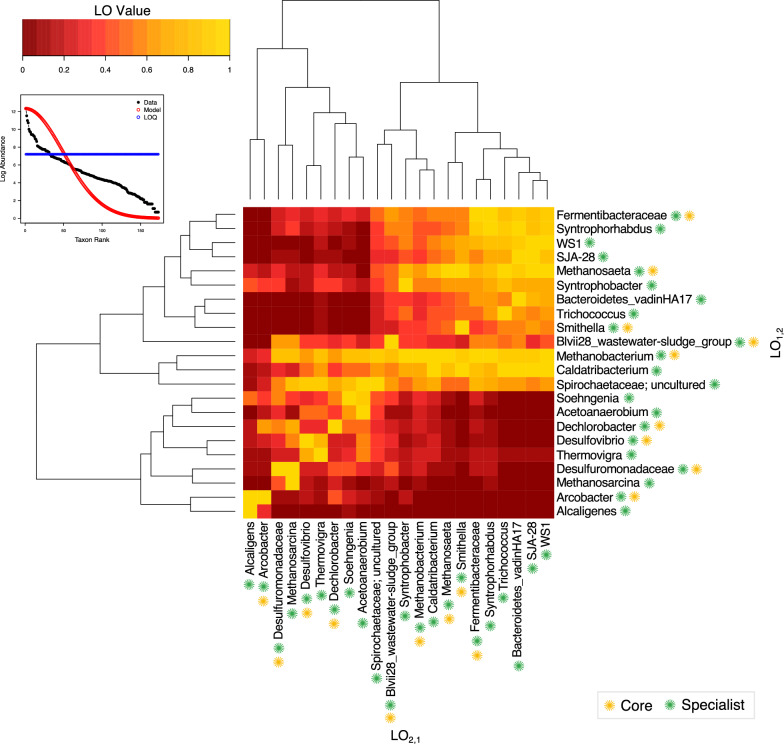
Specialists in anaerobic microbiomes. Levin’s ß_N_ identified specialists from all samples (Table 1). These selected genera were used to calculate Levin’s overlap with green and yellow asterisks respectively signifying specialists and those that were also a part of the core microbiome. Notably, the heatmap is not a symmetric map. LO_1,2_ and LO_2,1_ are not necessarily equal. When LO_1,2_ is equal to one, species 1 completely overlaps in all instances where species 2 exists. Conversely, when LO_2,1_ equals one, species 2 completely overlaps in all instances where species 1 exists

Our dataset yielded 22specialist genera, and no generalists (Table 1). Many of these specialists were also identified as members of the core community (Fig. 6). Levin’s overlap (LO) was then used to compare taxa pairs from amongst these specialists. *Methanobacterium* showed high overlap against nearly every other taxon indicating that it is present in all instances when the others are also present. Other groups with a broad overlap included *Spirochaetaceae* and Ca. *Caldatribacterium*.

**Table 1:** List of specialists in AD

Taxa	B_N_	p.val	p.adj	Description
PHOS-HE36	0.547	0.026*	0.028*	Specialist
Candidatus Caldatribacterium	0.241	0.000***	0.000***	Specialist
Trichococcus	0.100	0.000***	0.000***	Specialist
SJA-28	0.120	0.000***	0.000***	Specialist
Acetoanaerobium	0.369	0.000***	0.000***	Specialist
Soehngenia	0.508	0.008**	0.009**	Specialist
Thermovigra	0.548	0.026*	0.028*	Specialist
WS1	0.118	0.000***	0.000***	Specialist
Syntrophorhabdus	0.163	0.000***	0.000***	Specialist
Blvii28_wastewater-sludge_group	0.467	0.002**	0.002**	Specialist
Methanosarcina	0.269	0.000***	0.000***	Specialist
Bacteroidetes_vadinHA17	0.101	0.000***	0.000***	Specialist
Smithella	0.105	0.000***	0.000***	Specialist
Alcaligenes	0.101	0.000***	0.000***	Specialist
Fermentibacteraceae	0.206	0.000***	0.000***	Specialist
Dechlorobacter	0.505	0.007**	0.008**	Specialist
Spirochaetaceae, uncultured	0.548	0.026*	0.028*	Specialist
Syntrophobacter	0.262	0.000***	0.000***	Specialist
Desulfovibrio	0.532	0.017*	0.018**	Specialist
Desulfuromonadaceae	0.397	0.000***	0.000***	Specialist
Arcobacter	0.242	0.000***	0.000***	Specialist
Methanobacterium	0.355	0.000***	0.000***	Specialist
Methanosaeta	0.163	0.000***	0.000***	Specialist

Levin’s B_N_ results for genera recovered from individual granules. For all recovered genera the fifth quartile was 0.599 and the ninety-fifth quartile was 0.892

Significant P values are highlighted as p < 0.05 (*); p < 0.01 (**); p < 0.001 (***)

### Summary of returned taxa from all analyses

Many of the taxa highlighted by the various analyses were returned by more than one analytical tool (Table 2). Indeed, *Smithella* was returned by every analytical tool applied. Interestingly, most of the taxa identified as susceptible to environmental changes were not the most abundant groups or core groups. It was additionally notable that many of the specialists were also highly abundant, and were also usually either/both part of the stable subset and/or core.

**Table 2:** Significant taxa returned by all analytical tools

Taxa	Top-25	Resilient	Susceptible	Stable	Core	Specialist
PHOS-HE36	✱					✱
Candidatus Caldatribacterium	✱					✱
Trichococcus			✱	✱		✱
SJA-28						✱
Acetoanaerobium	✱			✱		✱
Soehngenia	✱					✱
Thermovigra	✱	✱				✱
WS1	✱		✱	✱		✱
Syntrophorhabdus						✱
Blvii28_wastewater-sludge_group	✱			✱	✱	✱
Methanosarcina	✱			✱		✱
Bacteroidetes_vadinHA17	✱		✱	✱		✱
Smithella	✱		✱	✱	✱	✱
Alcaligenes	✱			✱		✱
Fermentibacteraceae	✱			✱	✱	✱
Dechlorobacter	✱				✱	✱
Spirochaetaceae, uncultured	✱			✱		✱
Syntrophobacter			✱			✱
Desulfovibrio	✱	✱		✱	✱	✱
Desulfuromonadaceae	✱	✱		✱	✱	✱
Arcobacter	✱			✱	✱	✱
Methanobacterium	✱			✱	✱	✱
Methanosaeta	✱		✱		✱	✱
OPB41	✱	✱		✱	✱	
Lentimicrobium	✱	✱		✱	✱	
Wolinella	✱	✱				
Anaerovorax	✱	✱				
Syner-01	✱			✱	✱	
Mesotoga	✱			✱		
Trichloromonas				✱		
YC-ZSS-LKJ53			✱			
Oligoflexia			✱			
Candidatus_Raymondbacteria			✱			
LF045			✱			
Phaselicystis			✱			
Methanospirillum			✱			
Phycisphaerae			✱			
UASB-TL25			✱			
Methylomonas			✱			
WCHB1-81			✱			
Longilinea			✱			
Hydrogenispora			✱			
Moduliflexaceae			✱			
Acetobacterium			✱			
GWF2-44–16			✱			
Syntrophomonadaceae			✱			
MSBL9			✱			
Methanolinea			✱			
Methanomicrobium			✱			
Vermiphilaceae			✱			
Pla1 lineage			✱			

‘Top-25’ refers to the top-25 most abundant taxa across all samples; ‘Resilient’ denotes taxa whose abundances increased across all environmental conditions; ‘Susceptible’ refers to those whose abundances decreased across all environments; ‘Stable’ are those taxa which make up the subset community whose total abundances are preserved across all conditions; ‘Core’ refers to those taxa identified as part of the core microbiome; and ‘Specialist’ denotes specialists identified by Levin’s niche breadth

## Discussion

### Single granules are whole-ecosystem replicates

Sequencing of 16S rRNA (cDNA) from 16 individual, large granules indicated statistical similarity based on community structure. Previous studies sequenced 16S rRNA genes (DNA) of single anaerobic granules, concluding granules were heterogenic and that different granules may perform different functions [22]. This is indeed true, that differently sized single granules may indeed be functionally different [23]. However, our study went one step further, and focused on replicate granules from within one size fraction, investigating the active community in individual granules, and finding that they are statistically identical, or homogenic, and that at the depth of 16S rRNA, individual granules could be considered highly-replicated whole ecosystems.

### Single granules respond reproducibly to environmental cues

The individual granules yielded reproducible responses with respect to the structure of the active component of the microbiome, regardless of the environmental cue applied. This was especially true when testing for the pH or temperature effects. Moreover, VFA profiling demonstrated similar metabolisms across the eight replicate granules incubated under each condition.

The first effect investigated was the ‘plate effect’. The conditions imposed were as similar as possible to the source bioreactor conditions with respect to substrate, pH, temperature and metal supplementation. While the μBRs were placed on shakers during the incubations, what could not be replicated precisely were engineered conditions such as up-flow velocity, continuous feeding, metacommunity (granule-granule) interactions, and sheer forces. The ‘plate effect’ showed how single-granule incubation in μBRs induced changes in the community away from the ‘original’ granules. It is common in bioreactor systems for the inoculum to change, sometimes dramatically, at the first sampled timepoint [24–27], and this may be particularly true in the case of the active community [28], which may show greater sensitivity to environmental changes.

Next, we tested several pH and temperature effects. Temperature and pH gradients, or perturbations, are common in the environment as well as in controlled/engineered ecosystems. Moreover, anaerobic (especially methanogenic) communities are highly sensitive to pH changes [29]. Indeed, altering the pH in the μBRs induced significant shifts in the distribution of the active communities. At pH 4, a dramatic increase in relative abundance of *Arcobacter* was observed. This was also observed at the lower temperature. *Arcobacter* is a genus of typically heterotrophic bacteria growing on amino acids, sugars and short chain fatty acids (SCFA). They are often present in anaerobic wastewater treatment systems – but this may be one of the first instances where *Arcobacter* has been identified as both acid- and (potentially) low-temperature-tolerant within these types of communities. This is an example of how the μBRs provided a way to observe, in parallel, how different environmental conditions could result in similar community shifts.

The next two ‘effects’—substrate and cobalt—indicated more community overlap across conditions (Fig. 2). It is possible that these effects take more time to induce significant changes in the microbial community, or that they do not induce as strong a selective pressure as temperature and pH. Future experiments could test a wider range of substrates, or of trace metal supplementation or deprivation. We tested several substrates ranging from more complex organics (cellulose) to simpler compounds (acetate). We hypothesized that the simpler substrates would result in a more specialized community structure, but we observed only very minimal changes in diversity according to substrate. It’s probable that residual, complex substrates were embedded in the extracellular polymeric substances (EPS) matrix of the granular biofilms—which the higher-trophic-level hydrolyzers and fermenters could continue to utilize. It is worth noting, however, that incubating with the simpler substrates (acetate and VFA) specifically resulted in small increases in the relative abundance of the acetoclastic methanogens, as would be expected.

Trace metals are important in anaerobic environments to promote methanogenesis, specifically transition metals such as iron (Fe) nickel (Ni), cobalt (Co), molybdenum (Mo), selenium (Se) and tungsten (W) [30]. Cobalt, in particular, is involved in methyltransferase activity (MT I and MT II), which is required for methyl group transfer from methanol-containing compounds during methylotrophic methanogenesis [31–33]. Cobalt is additionally present as part of the membrane-associated sodium-ion translocating methyltransferase complex used by hydrogenotrophic and acetoclastic methanogens [34]. Given cobalt involvement in methanogenesis, we expected methanogenic archaea in Co-deprived granules (Conditions 8 and 9) to be negatively impacted, especially the methylotrophic methanogens. Indeed, according to GLLVM (Fig. 3), nearly all the methanogens were negatively associated with cobalt deprivation. There were minor differences between Condition 8 (acetate-fed) and Condition 9 (VFA-fed), where the methanogens supplied with VFA were slightly more resilient than when grown only on acetate. Relatedly, previous studies have shown that acetoclastic methanogens were more susceptible than hydrogenotrophic methanogens to cobalt deprivation [35], agreeing with our findings in which *Methanosaeta* was negatively associated with both Conditions 8 and 9. Moreover, other studies have shown that bioreactors suppled with methanol were more susceptible to cobalt supplementation than reactors supplied with acetate [36]. In our study, we show that methanogenic communities fed with acetate were more susceptible to cobalt limitations than methanogenic communities supplied with a VFA mixture. It may be deduced that methanol-supplied communities are the most susceptible to cobalt limitations, followed by acetate-supplied and, finally, VFA-supplied.

While each condition produced a unique community profile, which differed from the ‘original’, several groups were observed to have similar responses regardless of the condition applied. Taxa with positive responses to all conditions were identified as potentially *resilient*, while those which had negative responses to all conditions were identified as potentially *susceptible*. Resilience speaks to a microorganisms’ ability to adapt and endure during or after an environmental disturbance [37], and is an important concept for engineered communities, which can be subject to unexpected changes in pH, temperature, organic loading rates, etc. Resilience has been previously discussed in terms of whole-community resilience [38], but in this study, we were able to identify specific taxa which may be among the critical groups providing whole-community resilience. These taxa were: *Desulfuromonadaceae*, *OPB41*, *Wolinella*, *Anaerovorax*, *Lentimicrobium*, *Thermovigra* and *Desulfovibrio*.

Using the μBR set-up, we were able to demonstrate that individual granules are whole microbial communities, containing active taxa from across each of the trophic levels making up the metabolic chain of anaerobic digestion. Moreover, we showed that when subjected to specific environmental cues, individual granules responded reproducibly as whole microbial communities. In the same way that Rillig et al*.* [16] proposed that soil aggregates perform as evolutionary incubators containing complex microbial communities of importance to ecology, we propose individual anaerobic granules are much the same, and are whole-ecosystem biofilms. Severl aspects remain unexamined: (a) potential changes in the structural properties of the granules, and (b) how different layers of the granules (or different depths of the biofilm) may have experienced site-specific changes. These types of questions could be examined in the future using techniques such as fluorescent in situ hybridization (FISH). Indeed, countless tests could be conducted on this type of unique, niche microbiome and using this type of whole-community approach. High-throughput experiments could help answer ecological questions regarding microbial community strategies for environmental adaptation and community succession, as well as responses to changing climates.

### Imposed environmental conditions select for specialists

In our study we used two methods to address microbial community assembly: (i) Sloan’s model of neutrality, applied to ASVs identified within the core microbiome, and (ii) the micro-niche approach by incorporating Levin’s B_N_ metric and overlap. Each method has specific assumptions and limitations. Sloan’s model of neutrality [39] does not explicitly code for all possible environments and gives an account of species that are influenced only by the immediate environment—whether through selection or dispersal limitation. On the other hand, with the micro-niche approach, the different environments are coded in the definition of the Levin’s B_N_ metric, where species abundances are calculated for each environment separately. Although the micro-niche approach, in principle, should return generalist taxa, in our case we only observed specialist groups. This is because as a part of our experiment we have created artificial perturbations whose magnitude are more pronounced than the traditional variations/gradients frequently observed in natural ecosystems. Nonetheless, there may be generalist groups within the community, which we may not be picking up due to a combination of analytical and experimental limitations. Furthermore, there are also two schools of thought in terms of describing community assemblages. Some view assemblages in terms of stochasticity vs. determinism whilst others consider them in terms of neutrality vs. niche differentiation, with stochasticity often loosely associated with neutrality [18]. However, all four processes are different, as discussed by Tucker et al. [40], where neutrality is a process in which all species have identical growth rates, whilst niche processes are those where under certain conditions some species have optimal growth rates. In our analyses some of the neutral species are also returned as specialist species through the micro-niche approach. Again, the discrepancies may have arisen as a result of different assumptions in the two approaches. Since the perturbations applied in this study are pronounced, it is more appropriate to give more weightage to micro-niche outcomes, given that we were artificially creating such strong niche environments.

The taxa identified as specialists spanned the entire trophic chain required for complete anaerobic degradation of organic matter. These included organisms which oxidize complex polysaccharides (such as hemicellulose), proteins, and aromatics, as well as those using simpler by-products, such as propionate, butyrate, malate and fumarate. Several methanogenic archaea were also identified as specialists, namely: *Methanosaeta*, *Methanobacterium*, and *Methanosarcina*. From among this group of specialists, we would expect minimal substrate redundancy, given that each specialist must occupy a specific niche and is restricted by environmental parameters or intense competition between taxa [41]. Indeed, while we were somewhat limited by genus-level resolution, and many uncultured groups and literature gaps, the organisms which showed high overlap generally did not occupy the same niche (substrate preference). Take the methanogenic archaea, for example; heterotrophic *Methanosaeta* preferentially oxidizes acetate to produce methane, while autotrophic *Methanobacterium* will preferentially utilize H_2_ and CO_2_ as methanogenic substrates. *Methanosarcina*, in contrast, has a versatile metabolism and can choose from all the methanogenic pathways including carboxydotrophic (CO) and methylotrophic (methanol, methylamines, etc.) methanogenesis [42]. Each of these three archaea may then be said to occupy a specific niche in terms of substrate preference. In cases where two competing specialists did have a high overlap, we would hypothesize that they were not spatially juxtaposed within the granule.

## Conclusions

In this study, entire communities of anaerobic microorganisms were investigated in the form of anaerobic granules. Individual granules were highly replicated microbial biofilms and could be exploited as whole ecosystems, producing replicated responses in the active microbiome to various environmental cues. Furthermore, specialist taxa were identified spanning the entire trophic chain for the complete oxidation of complex organic matter. Overall, the study demonstrated that anaerobic granules are complex niche communities, flexibly shifting their active communities in response to environmental pressures. We conclude that there is potential for many high-throughput experiments using these unique whole-microbial-community biofilms.

## Methods

### Source of biomass and size fractionation

Methanogenic granules were sourced from a lab-scale UASB treating synthetic VFA-rich wastewater at 37 °C (details in Additional file 1). The biomass was size-fractionated using a range of stainless-steel sieves into three size fractions: small (S; Ø, 0.4–0.8 mm), medium (M; Ø, 0.8–1.2 mm) and large (L; Ø, 1.2–2.0 mm).

### Set-up of micro-batch reactors (μBRs)

Large granules (Ø, 1.2–2.0 mm) were sampled, and single, large granules were transferred into individual wells of 48-well plates (the μBRs). In an anaerobic chamber, anaerobic basal medium (details in Additional file 1) and corresponding substrates were added in 1-ml aliquots to each granule. All μBRs were then wrapped in parafilm (Parafilm M Wrapping Film, Fisher Scientific) and operated as 48-h batch incubations, with fresh medium supplied at the start of each 48-h cycle. The experiment lasted for a total of 42 days for all conditions (to be sure the communities had sufficient time to adapt). μBRs at 37 °C were placed inside an anaerobic box (BD GasPak EZ) and on a shaker inside a temperature-controlled room, while μBRs at 23 °C were incubated on a shaker in the temperature-controlled anaerobic chamber.

Nine different environmental conditions were simultaneously tested (Fig. 3) as factors which affect growth and activity, and factors which are widely known—across a wide range of community types (soil, sediment, stream, etc.) —to be strong influencers on microbiome structure and function. These included: (i) the pH effect (4,7, and 10); (ii) the temperature effect (37 °C and 23 °C); (iii) the substrate effect (VFA-mixture, acetate, cellulose, or glucose); and (iv) the cobalt effect (supplied or deprived [43]).

### Sugar and volatile fatty acid profiling

Sugar and VFA concentrations were monitored during the μBR trial as a means of targeted metabolomic profiling. During the μBR trial, liquor samples (*n* = 8) were collected for each relevant environmental condition: sugars were measured from single-granule incubations fed with cellulose or glucose, whilst VFA were monitored in all incubations (all conditions). Samples were collected on days 38 and 39 at hours: 0, 4, 8, 12, 20, 24, 28, 32, 36, 40, 42 and 48.

Sugars were measured using the Dubois method [44] with glucose as the standard. VFA profiling was measured using gas chromatography [14]. A flame ion detector (FID) was used and VFA were identified by assigning retention times and spectra to the relevant compounds (acetic, propionic, and butyric acids). Standard VFA calibration curves were used for comparison of relative VFA concentrations, which were expressed as mg l^−1^. The internal standard used was 2-Ethylbutyric acid.

### DNA/RNA co-extraction and cDNA synthesis

Genomic DNA and RNA were co-extracted from each sample: (i) from single-granule replicates (*n* = 16) before treatment, and (ii) from single granules (*n* = 3) from each treatment condition (n = 43 total). Nucleic acids were co-extracted following the method previously described [45], which is based on bead beating in 5% (w/v) cetyl trimethylammonium bromide (CTAB) extraction buffer, followed by phenol–chloroform extraction. Purity of nucleic acids was assessed using a nanodrop (Thermo Fisher Scientific, Waltham, MA, USA). Concentrations were determined using a Qubit fluorometer (Invitrogen, Carlsbad, CA, USA) and normalised to 5 ng DNA µl^−1^ before storage at – 80 °C.

cDNA was subsequently synthesized from all RNA extracts as follows: samples were defrosted on ice and RNA purification was achieved through a DNase treatment using the TurboDNase kit (AMBION—Invitrogen, Carlsbad, CA, USA) following the manufacturer’s recommendations. DNA removal was verified by 16S rRNA gene PCR (details in Additional file 1), using primer pair 338f and 805r. Once gel electrophoresis confirmed the absence of any residual DNA (absence of DNA bands) from the PCR products, cDNA was synthesized using a master-mix of MgCl_2_ (50 mM), Random Primer Mix (60 mM; BioLabs, MA, USA) and dNTPs (10 mM). M-MuLV reverse transcriptase (Biolabs, MA, USA) was used to catalyze synthesis. Concentrations were determined using a Qubit (Invitrogen, Carlsbad, CA, USA), and cDNA was stored at -20ºC prior to sequencing.

### Amplicon sequencing, bioinformatics and statistical analysis

Amplification of 16S rRNA gene sequences from cDNA from (i) the 16 single-granule replicates prior to treatment and (ii) the single granules sampled from the various incubations was performed by the Research and Testing Laboratory (Lubbock, Texas), using universal bacterial and archaeal primer set: forward primer 515F and reverse primer 806R. The resulting amplicon libraries of short inserts were sequenced on the Illumina MiSeq platform. Statistical analyses were performed in R using the data generated from the bioinformatics. Details are described further in Supplementary Methods (Additional file 1). An ASV table was generated for this study by matching the original barcoded reads against clean ASVs (a total of 1,685 ASVs for *n* = 43 samples).

## Supplementary Information

Supplemental material 1: Figure S1. Metabolic profiles of single granules. Figure S2. Sugar and VFA profiles of single granules.

## Data Availability

The sequencing data from this study are available on the European Nucleotide Archive under the study Accession Number PRJEB29753. Additional data are provided as supplementary materials.

## Abbreviations

μBR: Micro-batch reactor
AD: Anaerobic digestion
ANOVA: Analysis of variance
ASV: Amplicon sequencing variant
cDNA: Complementary DNA
CI: Confidence interval
CLR: Centralized log transformation
CTAB: Cetyl trimethylammonium bromide
DNA: Deoxyribonucleic acid
EPS: Extracellular polymeric substances
EQO: Ensemble quotient optimization
FID: Flame ion detector
GLLVM: Generalized linear latent variable model
LO: Levin’s overlap
PCoA: Principle coordinate analysis
PERMANOVA: Permutational analysis of variance
RNA: Ribonucleic acid
UASB: Upflow anaerobic sludge bed
VFA: Volatile fatty acids
