# Supplementary material for: Individual methanogenic granules are whole-ecosystem replicates with reproducible responses to environmental cues
Source: Environ Microbiome. 2024 Sep 9;19:68. doi: 10.1186/s40793-024-00615-z (PMC11386378; doi:10.1186/s40793-024-00615-z)
Supplement: Supplementary file 1 — Supplemental material 1: Figure S1. Metabolic profiles of single granules. Figure S2. Sugar and VFA profiles of single granules. [file 40793_2024_615_MOESM1_ESM.docx]

**INDIVIDUAL METHANOGENIC GRANULES ARE WHOLE-ECOSYSTEM REPLICATES WITH REPRODUCIBLE RESPONSES TO ENVIRONMENTAL CUES**

**Trego et al. Additional File 1**

**Supplemental Materials and Methods:**

*Information on sludge sources*

**Table S1.** Operational parameters of the anaerobic digester from which the granules were sampled

|  | **Source** |
| --- | --- |
| *Scale* | Lab-Scale |
| *Wastewater* | Synthetic VFA mix |
| *pH* | 6.5-7.5 |
| *Temperature* | 37 |

The granules were sampled from a set of triplicate mesophilic, lab-scale UASB reactors treating a synthetic volatile fatty acid (VFA) wastewater at 37°C. Samples were taken from the UASB reactors after 112 days of operation and from a combination of reactor sampling ports. These samples were pooled into a single biomass sample before being size-fractionated. The biomass was originally sourced from a full-scale (1500 m^3^), internal circulation (IC) bioreactor at Carbery Milk Products (Ballineen, Co. Cork, Ireland).

*Anaerobic basal medium and stock solutions*

The anaerobic basal medium for each individual granule consisted of (in grams per litre of distilled water): KH_2_PO_4_, 0.27, K_2_HPO_4_, 0.27, trace metal solution (H_3_BO_3_, 0.05 g, CoCl_2_.6H_2_O, 0.5 g, CuCl_2_.2H_2_O, 0.03 g, MnCl_2_.4H_2_O, 0.5 g, NaMo_4_.2H_2_O, 0.01 g, NiCl_2_.6H_2_O, 0.05 g, Na_2_SeO_3_, 0.05 g, ZnCl_2_, 0.05 g, in 1000ml of distilled water), 1 ml, trace element solution (NH_4_Cl, 132.5 g, MgCl_2_.2H_2_O, 25 g, CaCl_2_, 14.15 g, FeCl_2_.4H_2_O, 5 g, in 1000ml of distilled water), 4 ml and L-cysteine-HCl.H_2_O, 0.622. After boiling and sparging the medium with N_2_ gas, 3.05 g of NaHCO3 was added to the medium.

Sterile stock solutions of a VFA mixture (acetic acid, 15 mM, propionic acid, 9 mM and butyric acid, 6 mM), acetate (30 mM), cellulose (0.0211g/l or 8.3 µM), glucose (0.2346 g/l or 1.3 mM) and 2-bromoethanesulphanate (BES) (50 mM) were stored at 4°C for the duration of the trial. A trace metal solution (as described above but without CoCl_2_.6H_2_O) was made up for granules deprived of cobalt. Granules that were not deprived of cobalt had a Co concentration of 2.11µM. pHs were altered with 5M HCl to lower the solutions to pH 4 and with 8M NaOH to raise the solutions to pH 10 using a pH meter for reference (HI-207 Bench pH Meter, Hanna, UK).

*16S rRNA PCR conditions to check DNAse treatment*

The LightCycler 480 Probe Master kit (Roche Diagnostics) was used to prepare 20 μl reaction mixtures containing 2 μl of PCR-grade water, 1 μl of each primer (with a final concentration of 10 mM), 10 μl of 2x LightCycler® 480 Probes Master, and 5 μl of template DNA. The thermal cycling program (LightCycler 480 instrument, Roche, Mannheim, Germany), used for each reaction included pre-denaturation at 94°C for 10 min, 45 cycles of denaturation at 95°C for 10 s and simultaneous annealing and extension at 60°C for 30 s. All samples were analysed in duplicate.

*Bioinformatics*

We have used DADA2 algorithm [1] in the QIIME2 framework [2] to generate Amplicon Sequencing Variants (ASVs) as described previously by the authors [3]. This resulted in a final list of 1,685 ASVs for n=43 samples after removing for standard contaminations including as *Mitochondria* and *Chloroplasts*, as well as any ASVs that were unassigned at all levels, as per recommendations given at <https://docs.qiime2.org/2022.8/tutorials/filtering/>. Note that the taxonomy for the ASVs was assigned using the SSU Ref NR database release v.138 [4] with the rooted phylogenetic tree also generated using the QIIME2 framework. The summary statistics of final sample-wise ASVs are: [1^st^ Quartile: 11,656; Median: 15,735; Mean: 14985; 3^rd^ Quartile: 19,046].

*Statistical analyses*

The vegan package [5] was used for alpha (Chao1 richness, Shannon entropy) and beta diversity analyses using Bray-Curtis distance in the Principal Coordinate Analysis. Analysis of variance was also performed using Vegan’s adonis2() function against sources of variation using Bray-Curtis distance.

To find the relationship between individual microbial genera and all conditions, we have used *Generalised Linear Latent Variable Model* (GLLVM) [6], which extends the basic generalized linear model that regresses the mean abundances $\mu_{ij}$ (for $i$-th sample and $j$-th genus) against the covariates $x_{i}$ as above by incorporating latent variables $u_{i}$ as $g\left( \mu_{ij} \right)=\eta_{ij}=\alpha_{i}+\beta_{0j}+\boldsymbol{x}_{i}^{T}\boldsymbol{\beta}_{j}+\boldsymbol{u}_{i}^{T}\boldsymbol{\theta}_{j}$, where $\boldsymbol{\beta}_{j}$ are the genus specific coefficients associated with individual covariate (a 95% confidence interval of these whether positive or negative, and not crossing 0 boundary gives directionality with the interpretation that an increase or decrease in that particular covariate causes an increase or decrease in the abundance of the genus/gene), and $\boldsymbol{\theta}_{j}$ are the corresponding coefficients associated with latent variable. $\beta_{0j}$ are genus-specific intercepts, whilst $\alpha_{i}$ are optional sample effects which can either be chosen as fixed effects or random effects. To model the distribution of individual genera, we have used *Negative Binomial distribution*. Additionally, the approximation to the log-likelihood is done through *Laplace Approximation* (LA) with final sets of parameters in glvmm() function being family = 'negative.binomial', and method="LA”. For convergence of GLLVM algorithm, we have used control.start=list(n.init = 4, jitter.var = 0.1). This, we did for top 100 most abundant genera in our dataset.

We next wanted to see if we can find a minimal subset of species that either remain stable across all conditions. For this purpose we incorporated the Ensemble Quotient Optimisation (EQO) approach of [7]. The approach uses a relative abundance table, called community matrix $\boldsymbol{M}$ (*P* ASVs over *n* samples), where the goal is to obtain a vector $\boldsymbol{x}\in\left( 0,1 \right)^{P}$ where the *i^th^* position in the vector is either 0 or 1, i.e., a subset of species with values 1 belong to an ensemble which we are interested in recovering. This ensemble is recovered in the context of a phenotype/predictor variable $\boldsymbol{y}$ by optimizing an *Ensemble Quotient* $EQ=\frac{\boldsymbol{x}^{T}\boldsymbol{Qx}}{\boldsymbol{x}^{T}\boldsymbol{Px}}$, through a genetic algorithm (an optimization algorithm), where $\boldsymbol{P}$ and $\boldsymbol{Q}$ are algebraic transformations of the community matrix that capture the covariance between species, and the covariance between species and $\boldsymbol{y}$. The choice of $\boldsymbol{y}$ dictates what ensemble we recover, and is considered uniform i.e., consisting of 1s, with $\boldsymbol{Q=}\boldsymbol{M}^{T}\boldsymbol{1}\boldsymbol{1}^{T}\boldsymbol{M}$, and $\boldsymbol{P=}\boldsymbol{M}^{T}\boldsymbol{M-}\frac{2}{n}\boldsymbol{M}^{T}\boldsymbol{1}\boldsymbol{1}^{T}\boldsymbol{M+}\frac{\boldsymbol{1}}{n^{2}}\boldsymbol{M}^{T}\boldsymbol{1}\boldsymbol{1}^{T}\boldsymbol{1}\boldsymbol{1}^{T}\boldsymbol{M}$. Quality of fit is returned as Coefficient of Variation (CV) which approaching 0 means we are able to find a stable subset whose collated abundance doesn’t change across all samples. To optimize the EQ to obtain $\boldsymbol{x}$, we followed the genetic algorithm optimization located at <https://github.com/Xiaoyu2425/Ensemble-Quotient-Optimization>. In the genetic algorithm, we have used the following parameterizations: a population size of 100 solutions, maximum of 600 generations, and a maximum 20 taxa (ASVs collated at genus level) to be returned as an ensemble.

To identify core microbiome, we have used a model which incorporates a condition-specific occupancy model, where each condition and replicates across those conditions are used to derive the ranking of ASVs [8]. The approach first ranks the ASVs by getting a score based on condition specific occupancy as well as replicate consistency across these different conditions. After ranking the ASVs, the Bray-Curtis similarity is calculated between the samples using all the ASVs. The subset of core taxa is then constructed by taking the highly ranked ASVs as core and adding an ASV incrementally to this subset, calculating the Bray-Curtis similarity for the subset, and calculating its contribution using C=1 – BC (subset)/BC(all). The approach stops when addition of an ASV did not cause more than 2% increase in the explanatory value by Bray-Curtis distance. Independently, a neutral model [9] was fitted to the “S” shaped abundance-occupancy distributions inform the ASVs that are likely selected by the environment. These were obtained as those that fall outside the 95% confidence interval of the fitted model, and were inferred to be deterministically assembled, rather than neutrally selected (represented by yellow colour), with those that are above the model selected by the host environment (represented by blue colour), and those points below the model were dispersal limited (represented by red colour).

To identify the roles of microbes within the context of all these conditions, we have used the R’s MicroNiche package [10]. The aim is to determine *generalist* (that should exist in majority of the conditions) and *specialist* (that should exist in some conditions) microbial species. Before, applying these approaches, we filtered out genera by using the limit of quantification (LOQ) approach as per author’s instruction. Briefly, LOQ filters out microbes that fall below a “decision boundary”, calculated from the distribution of microbes with 95% certainty that these microbes will fall within a null distribution where the mean microbial abundance is zero. To calculate the standard deviation of the null distribution, the lognormal rank distribution of the microbes with the dataset was fitted with $S\left( R \right)=S_{0}e^{-a^{2}R^{2}}$ where log abundance of microbe $S$ at rank $R$ is dependent on coefficient $a$ and rank $R$ calculated as $a=\sqrt{\frac{\ln S_{0}}{S_{m}}/R^{2}}$ where $S_{m}$ is the lowest taxon abundance of $S$. To calculate LOQ, we fit the above log normal model to data, and LOQ is then determined as the overlap between the null hypothesis (i.e., a microbe’s mean abundance is zero) and where the microbe falls within 1.65 standard deviation of the above model.

After filtering out the genera, we then calculated the niche breadth as Levins’ $B_{N}=\frac{1}{R}\sum_{i=1} p_{i}^{2}$, where $p_{i}$ is the proportional abundance of a genus in the $i$-th condition, with total number of conditions being $R$ (10 in this case). If $B_{N}$ approaches 1 for a given genus, then it is considered as a “generalist”, whilst if it approaches $1/R$, then it can be tagged as a “specialist”. To derive the p-value for Levins’ $B_{N}$ i.e., if it we can call a genus a generalist or a specialist with great certainty, a null modelling approach is used, where a random normal distribution of 999 possible niche breadths were produced for a genus, and allows a p-value to be assigned depending on whether a genus’s $B_{N}$ is greater or lower than the mean of the null model. As per author’s recommendation, after applying null modelling, the 5^th^ Quantile and 95^th^ Quantile were obtained to tag the genera as specialist if its $B_{N}$ < 5^th^ Quantile, and generalist, if its $B_{N}$ > 95^th^ Quantile. Those that fell in the inter-range were tagged as undecided. In the second step, we then calculated the overlap of the specialists using Levins’ Overlap formula $LO_{i,j}=\frac{\sum_{i,j=1} (p_{ir})(p_{jr})}{\sum_{i=1} (p_{ir}^{2})}$, where $p_{i}$ is the proportional abundance of genus $i$ in the $r$-th condition, and $p_{j}$ is the abundance of genus $j$ in the $r$-th condition, where $i$ and $j$ were selected after tagging an individual genus as specialists only (since no generalist was selected).

**Trego et al. Supplemental Figures**

**Figure S1.** Metabolic profiles of single granules fed with **(a)** VFA at pH 7 and 37°C; **(b)** VFA at pH 4 and 37°C; **(c)** VFA at pH 10 and 37°C; **(d)** VFA at pH 7 and 23°C; **(e)** VFA at pH 7 and 37°C but deprived of cobalt; **(f)** Acetate at pH 7 and 37°C and; **(g)** Acetate at pH 7 and 37°C but deprived of cobalt. All conditions compare the VFA consumption for each condition both with and without the addition of BES.

**Figure S2.** Sugar and VFA profiles from single granule experiments supplied with **(a)** cellulose; **(b)** cellulose and BES; **(c)** glucose and; **(d)** glucose and BES where dashed lines represent sugar consumption and continuous lines represent VFA accumulation.

Table S1. List of returned core ASVs sorted with respect to ‘ASV_occ” which represents the occupancy value of a given ASV.

|  | **ASV** | **ASV_occ** | **ASV_rel** | **Neutral** | **Kingdom** | **Phylum** | **Class** | **Order** | **Family** | **Genus** | **ASV** |
| --- | --- | --- | --- | --- | --- | --- | --- | --- | --- | --- | --- |
| 18 | ASV_20 | 0.29268293 | 0.01471119 | Below | Bacteria | Campilobacterota | Campylobacteria | Campylobacterales | Arcobacteraceae | Arcobacter |  |
| 25 | ASV_28 | 0.29268293 | 0.01304986 | Below | Bacteria | Campilobacterota | Campylobacteria | Campylobacterales | Arcobacteraceae | Arcobacter |  |
| 19 | ASV_21 | 0.31707317 | 0.01433077 | Below | Bacteria | Campilobacterota | Campylobacteria | Campylobacterales | Arcobacteraceae | Arcobacter |  |
| 26 | ASV_30 | 0.31707317 | 0.01140298 | Below | Bacteria | Campilobacterota | Campylobacteria | Campylobacterales | Arcobacteraceae | Arcobacter |  |
| 70 | ASV_161 | 0.31707317 | 0.00150724 | no | Bacteria | Spirochaetota | Spirochaetia | Spirochaetales | Spirochaetaceae | uncultured | uncultured_Treponema |
| 74 | ASV_180 | 0.31707317 | 0.00104495 | Above | Bacteria | Bacteroidota | Bacteroidia | Sphingobacteriales | Lentimicrobiaceae | Lentimicrobium | |
| 79 | ASV_226 | 0.31707317 | 0.00187321 | no | Bacteria | Bacteroidota | Bacteroidia | Bacteroidales | Williamwhitmaniaceae | Blvii28_wastewater-sludge_group | uncultured_bacterium |
| 82 | ASV_246 | 0.31707317 | 0.00156021 | no | Bacteria | Desulfobacterota | Desulfovibrionia | Desulfovibrionales | Desulfovibrionaceae | Desulfovibrio | Desulfovibrio_oxamicus |
| 83 | ASV_253 | 0.31707317 | 0.00176245 | no | Bacteria | Actinobacteriota | Coriobacteriia | OPB41 | OPB41 | OPB41 | uncultured_bacterium |
| 47 | ASV_67 | 0.34146341 | 0.00375605 | no | Bacteria | Desulfobacterota | Desulfuromonadia | Desulfuromonadia | Desulfuromonadaceae | Desulfuromonadaceae | |
| 59 | ASV_97 | 0.34146341 | 0.00212361 | no | Bacteria | Synergistota | Synergistia | Synergistales | Synergistaceae | Syner-01 |  |
| 62 | ASV_113 | 0.34146341 | 0.00173356 | no | Bacteria | Synergistota | Synergistia | Synergistales | Synergistaceae | Syner-01 | metagenome |
| 75 | ASV_186 | 0.34146341 | 0.0015891 | no | Bacteria | Bacteroidota | Bacteroidia | Sphingobacteriales | Lentimicrobiaceae | Lentimicrobium | |
| 76 | ASV_199 | 0.34146341 | 0.00185395 | no | Bacteria | Actinobacteriota | Coriobacteriia | OPB41 | OPB41 | OPB41 | uncultured_bacterium |
| 78 | ASV_221 | 0.34146341 | 0.0020273 | no | Bacteria | Actinobacteriota | Coriobacteriia | OPB41 | OPB41 | OPB41 | uncultured_bacterium |
| 80 | ASV_231 | 0.34146341 | 0.00169985 | no | Bacteria | Fermentibacterota | Fermentibacteria | Fermentibacterales | Fermentibacteraceae | Fermentibacteraceae | uncultured_bacterium |
| 65 | ASV_150 | 0.36585366 | 0.00191655 | no | Bacteria | Desulfobacterota | Syntrophia | Syntrophales | Smithellaceae | Smithella |  |
| 67 | ASV_153 | 0.36585366 | 0.00156984 | no | Bacteria | Bacteroidota | Bacteroidia | Sphingobacteriales | Lentimicrobiaceae | Lentimicrobium | |
| 71 | ASV_169 | 0.36585366 | 0.00135314 | Above | Bacteria | Bacteroidota | Bacteroidia | Sphingobacteriales | Lentimicrobiaceae | Lentimicrobium | |
| 77 | ASV_201 | 0.36585366 | 0.00198396 | no | Bacteria | Bacteroidota | Bacteroidia | Bacteroidales | Rikenellaceae | uncultured | uncultured_anaerobic |
| 14 | ASV_16 | 0.3902439 | 0.01693112 | Below | Bacteria | Campilobacterota | Campylobacteria | Campylobacterales | Arcobacteraceae | Arcobacter |  |
| 21 | ASV_24 | 0.3902439 | 0.01365661 | Below | Bacteria | Campilobacterota | Campylobacteria | Campylobacterales | Arcobacteraceae | Arcobacter |  |
| 42 | ASV_56 | 0.3902439 | 0.0049262 | Below | Bacteria | Desulfobacterota | Desulfuromonadia | Desulfuromonadia | Desulfuromonadaceae | Desulfuromonadaceae | |
| 44 | ASV_58 | 0.3902439 | 0.00200804 | no | Bacteria | Synergistota | Synergistia | Synergistales | Synergistaceae | Syner-01 | uncultured_bacterium |
| 61 | ASV_106 | 0.3902439 | 0.0048925 | Below | Bacteria | Proteobacteria | Gammaproteobacteria | Burkholderiales | Rhodocyclaceae | Dechlorobacter | |
| 68 | ASV_157 | 0.3902439 | 0.00143019 | Above | Bacteria | Bacteroidota | Bacteroidia | Sphingobacteriales | Lentimicrobiaceae | Lentimicrobium | |
| 69 | ASV_158 | 0.3902439 | 0.00303855 | no | Bacteria | Desulfobacterota | Syntrophia | Syntrophales | Smithellaceae | Smithella |  |
| 72 | ASV_171 | 0.3902439 | 0.00268702 | no | Bacteria | Desulfobacterota | Syntrophia | Syntrophales | Smithellaceae | Smithella |  |
| 73 | ASV_178 | 0.3902439 | 0.0024944 | no | Bacteria | Desulfobacterota | Syntrophia | Syntrophales | Smithellaceae | Smithella |  |
| 81 | ASV_236 | 0.3902439 | 0.00247996 | no | Bacteria | Desulfobacterota | Syntrophia | Syntrophales | Smithellaceae | Smithella |  |
| 33 | ASV_43 | 0.41463415 | 0.00633231 | Below | Bacteria | Desulfobacterota | Desulfuromonadia | Desulfuromonadia | Desulfuromonadaceae | Desulfuromonadaceae | |
| 36 | ASV_46 | 0.41463415 | 0.00557147 | Below | Bacteria | Desulfobacterota | Desulfuromonadia | Desulfuromonadia | Desulfuromonadaceae | Desulfuromonadaceae | |
| 37 | ASV_47 | 0.41463415 | 0.00523439 | Below | Bacteria | Desulfobacterota | Desulfuromonadia | Desulfuromonadia | Desulfuromonadaceae | Desulfuromonadaceae | |
| 43 | ASV_57 | 0.41463415 | 0.00519587 | Below | Bacteria | Desulfobacterota | Desulfuromonadia | Desulfuromonadia | Desulfuromonadaceae | Desulfuromonadaceae | |
| 52 | ASV_78 | 0.41463415 | 0.00291335 | no | Bacteria | Spirochaetota | Spirochaetia | Spirochaetales | Spirochaetaceae | uncultured | uncultured_Treponema |
| 54 | ASV_85 | 0.41463415 | 0.00291335 | no | Bacteria | Spirochaetota | Spirochaetia | Spirochaetales | Spirochaetaceae | uncultured | uncultured_Treponema |
| 66 | ASV_152 | 0.41463415 | 0.00425204 | no | Bacteria | Proteobacteria | Gammaproteobacteria | Burkholderiales | Rhodocyclaceae | Dechlorobacter | |
| 41 | ASV_54 | 0.43902439 | 0.00182987 | Above | Bacteria | Synergistota | Synergistia | Synergistales | Synergistaceae | Syner-01 | uncultured_bacterium |
| 55 | ASV_89 | 0.43902439 | 0.00290853 | no | Bacteria | Spirochaetota | Spirochaetia | Spirochaetales | Spirochaetaceae | uncultured | uncultured_Treponema |
| 56 | ASV_91 | 0.43902439 | 0.0021814 | no | Bacteria | Bacteroidota | Bacteroidia | Sphingobacteriales | Lentimicrobiaceae | Lentimicrobium | Lentimicrobium_saccharophilum |
| 48 | ASV_71 | 0.46341463 | 0.00377531 | no | Bacteria | Spirochaetota | Spirochaetia | Spirochaetales | Spirochaetaceae | uncultured | uncultured_Treponema |
| 49 | ASV_72 | 0.46341463 | 0.0020899 | Above | Bacteria | Synergistota | Synergistia | Synergistales | Synergistaceae | Syner-01 | uncultured_bacterium |
| 51 | ASV_77 | 0.46341463 | 0.00284111 | no | Bacteria | Bacteroidota | Bacteroidia | Sphingobacteriales | Lentimicrobiaceae | Lentimicrobium | Lentimicrobium_saccharophilum |
| 53 | ASV_80 | 0.46341463 | 0.00337081 | no | Bacteria | Synergistota | Synergistia | Synergistales | Synergistaceae | Syner-01 | uncultured_bacterium |
| 58 | ASV_95 | 0.46341463 | 0.00228252 | no | Bacteria | Bacteroidota | Bacteroidia | Sphingobacteriales | Lentimicrobiaceae | Lentimicrobium | Lentimicrobium_saccharophilum |
| 63 | ASV_116 | 0.46341463 | 0.00231623 | no | Bacteria | Bacteroidota | Bacteroidia | Sphingobacteriales | Lentimicrobiaceae | Lentimicrobium | |
| 64 | ASV_127 | 0.46341463 | 0.0043965 | no | Bacteria | Proteobacteria | Gammaproteobacteria | Burkholderiales | Rhodocyclaceae | Dechlorobacter | |
| 45 | ASV_63 | 0.48780488 | 0.00466135 | no | Bacteria | Spirochaetota | Spirochaetia | Spirochaetales | Spirochaetaceae | uncultured | uncultured_Treponema |
| 60 | ASV_101 | 0.48780488 | 0.00330821 | no | Bacteria | Synergistota | Synergistia | Synergistales | Synergistaceae | Syner-01 | uncultured_bacterium |
| 57 | ASV_94 | 0.51219512 | 0.00289408 | no | Bacteria | Synergistota | Synergistia | Synergistales | Synergistaceae | Syner-01 | uncultured_bacterium |
| 46 | ASV_64 | 0.53658537 | 0.0037705 | no | Bacteria | Bacteroidota | Bacteroidia | Sphingobacteriales | Lentimicrobiaceae | Lentimicrobium | Lentimicrobium_saccharophilum |
| 50 | ASV_73 | 0.53658537 | 0.00398719 | no | Bacteria | Spirochaetota | Spirochaetia | Spirochaetales | Spirochaetaceae | uncultured | uncultured_Treponema |
| 84 | ASV_513 | 0.58536585 | 0.00097272 | Above | Archaea | Halobacterota | |  |  |  |  |
| 10 | ASV_10 | 0.7804878 | 0.02570968 | Below | Archaea | Halobacterota | |  |  |  |  |
| 13 | ASV_14 | 0.7804878 | 0.02050418 | Below | Archaea | Halobacterota | |  |  |  |  |
| 1 | ASV_1 | 0.80487805 | 0.02465509 | Below | Archaea | Halobacterota | Methanosarcinia | Methanosarciniales | Methanosaetaceae | Methanosaeta | |
| 2 | ASV_2 | 0.80487805 | 0.02585896 | Below | Archaea | Halobacterota | |  |  |  |  |
| 3 | ASV_3 | 0.80487805 | 0.02938868 | Below | Archaea | Halobacterota | |  |  |  |  |
| 5 | ASV_5 | 0.80487805 | 0.03373703 | Below | Archaea | Halobacterota | |  |  |  |  |
| 15 | ASV_17 | 0.80487805 | 0.004488 | Above | Archaea | Euryarchaeota | Methanobacteria | Methanobacteriales | Methanobacteriaceae | Methanobacterium | archaeon_enrichment |
| 16 | ASV_18 | 0.80487805 | 0.01979149 | Below | Archaea | Halobacterota | |  |  |  |  |
| 6 | ASV_6 | 0.82926829 | 0.03230684 | Below | Archaea | Halobacterota | |  |  |  |  |
| 7 | ASV_7 | 0.82926829 | 0.02926348 | Below | Archaea | Halobacterota | |  |  |  |  |
| 9 | ASV_9 | 0.82926829 | 0.00768064 | no | Archaea | Euryarchaeota | Methanobacteria | Methanobacteriales | Methanobacteriaceae | Methanobacterium | archaeon_enrichment |
| 34 | ASV_44 | 0.82926829 | 0.00576891 | Above | Archaea | Euryarchaeota | Methanobacteria | Methanobacteriales | Methanobacteriaceae | Methanobacterium | |
| 39 | ASV_49 | 0.82926829 | 0.00531625 | Above | Archaea | Euryarchaeota | Methanobacteria | Methanobacteriales | Methanobacteriaceae | Methanobacterium | |
| 11 | ASV_11 | 0.85365854 | 0.02762622 | Below | Archaea | Halobacterota | |  |  |  |  |
| 17 | ASV_19 | 0.85365854 | 0.00600005 | Above | Archaea | Euryarchaeota | Methanobacteria | Methanobacteriales | Methanobacteriaceae | Methanobacterium | archaeon_enrichment |
| 20 | ASV_22 | 0.85365854 | 0.01059399 | no | Archaea | Euryarchaeota | Methanobacteria | Methanobacteriales | Methanobacteriaceae | Methanobacterium | archaeon_enrichment |
| 22 | ASV_25 | 0.85365854 | 0.00842703 | no | Archaea | Euryarchaeota | Methanobacteria | Methanobacteriales | Methanobacteriaceae | Methanobacterium | archaeon_enrichment |
| 4 | ASV_4 | 0.87804878 | 0.03942889 | Below | Archaea | Halobacterota | |  |  |  |  |
| 8 | ASV_8 | 0.87804878 | 0.00677534 | Above | Archaea | Euryarchaeota | Methanobacteria | Methanobacteriales | Methanobacteriaceae | Methanobacterium | archaeon_enrichment |
| 27 | ASV_35 | 0.87804878 | 0.00565334 | Above | Archaea | Euryarchaeota | Methanobacteria | Methanobacteriales | Methanobacteriaceae | Methanobacterium | archaeon_enrichment |
| 32 | ASV_42 | 0.87804878 | 0.00834517 | Above | Archaea | Euryarchaeota | Methanobacteria | Methanobacteriales | Methanobacteriaceae | Methanobacterium | |
| 35 | ASV_45 | 0.87804878 | 0.00659235 | Above | Archaea | Euryarchaeota | Methanobacteria | Methanobacteriales | Methanobacteriaceae | Methanobacterium | |
| 40 | ASV_51 | 0.87804878 | 0.00761804 | Above | Archaea | Euryarchaeota | Methanobacteria | Methanobacteriales | Methanobacteriaceae | Methanobacterium | |
| 28 | ASV_36 | 0.90243902 | 0.01097922 | no | Archaea | Euryarchaeota | Methanobacteria | Methanobacteriales | Methanobacteriaceae | Methanobacterium | |
| 31 | ASV_41 | 0.90243902 | 0.00743987 | Above | Archaea | Euryarchaeota | Methanobacteria | Methanobacteriales | Methanobacteriaceae | Methanobacterium | |
| 38 | ASV_48 | 0.90243902 | 0.00865336 | Above | Archaea | Euryarchaeota | Methanobacteria | Methanobacteriales | Methanobacteriaceae | Methanobacterium | |
| 12 | ASV_12 | 0.92682927 | 0.00881709 | Above | Archaea | Euryarchaeota | Methanobacteria | Methanobacteriales | Methanobacteriaceae | Methanobacterium | archaeon_enrichment |
| 23 | ASV_26 | 0.92682927 | 0.00972239 | Above | Archaea | Euryarchaeota | Methanobacteria | Methanobacteriales | Methanobacteriaceae | Methanobacterium | archaeon_enrichment |
| 24 | ASV_27 | 0.92682927 | 0.01060843 | no | Archaea | Euryarchaeota | Methanobacteria | Methanobacteriales | Methanobacteriaceae | Methanobacterium | archaeon_enrichment |
| 30 | ASV_38 | 0.92682927 | 0.01007873 | Above | Archaea | Euryarchaeota | Methanobacteria | Methanobacteriales | Methanobacteriaceae | Methanobacterium | |
| 29 | ASV_37 | 0.97560976 | 0.00991019 | Above | Archaea | Euryarchaeota | Methanobacteria | Methanobacteriales | Methanobacteriaceae | Methanobacterium | |

**References**

1. Callahan BJ, McMurdie PJ, Rosen MJ, Han AW, Johnson AJA, Holmes SP. DADA2: High-resolution sample inference from Illumina amplicon data. Nat Methods [Internet]. 2016;13:581–3. Available from: https://doi.org/10.1038/nmeth.3869

2. Bolyen E, Rideout JR, Dillon MR, Bokulich NA, Abnet CC, Al-Ghalith GA, et al. Reproducible, interactive, scalable and extensible microbiome data science using QIIME 2. Nat Biotechnol. Nature Publishing Group; 2019;37:852–7.

3. Trego AC, McAteer PG, Nzeteu C, Mahony T, Abram F, Ijaz UZ, et al. Combined Stochastic and Deterministic Processes Drive Community Assembly of Anaerobic Microbiomes During Granule Flotation [Internet]. Front. Microbiol. . 2021. p. 1165. Available from: https://www.frontiersin.org/article/10.3389/fmicb.2021.666584

4. Quast C, Pruesse E, Yilmaz P, Gerken J, Schweer T, Yarza P, et al. The SILVA ribosomal RNA gene database project: improved data processing and web-based tools. Nucleic Acids Res [Internet]. 2012/11/28. Oxford University Press; 2013;41:D590–6. Available from: https://www.ncbi.nlm.nih.gov/pubmed/23193283

5. Oksanen J, Blanchet F, Kindt R, Legendre P, Minchin PR, O’hara R, et al. Vegan: community ecology package. R Package version 2.2-1. 2015.

6. Niku J, Hui FKC, Taskinen S, Warton DI. gllvm: Fast analysis of multivariate abundance data with generalized linear latent variable models in r. Methods Ecol Evol [Internet]. John Wiley & Sons, Ltd; 2019;10:2173–82. Available from: https://doi.org/10.1111/2041-210X.13303

7. Shan X, Goyal A, Gregor R, Cordero OX. Annotation-free discovery of functional groups in microbial communities. Nat Ecol Evol [Internet]. 2023;7:716–24. Available from: https://doi.org/10.1038/s41559-023-02021-z

8. Shade A, Stopnisek N. Abundance-occupancy distributions to prioritize plant core microbiome membership. Curr Opin Microbiol [Internet]. 2019;49:50–8. Available from: https://www.sciencedirect.com/science/article/pii/S1369527419300426

9. Burns AR, Stephens WZ, Stagaman K, Wong S, Rawls JF, Guillemin K, et al. Contribution of neutral processes to the assembly of gut microbial communities in the zebrafish over host development. ISME J [Internet]. 2016;10:655–64. Available from: https://doi.org/10.1038/ismej.2015.142

10. Finn DR, Yu J, Ilhan ZE, Fernandes VMC, Penton CR, Krajmalnik-Brown R, et al. MicroNiche: an R package for assessing microbial niche breadth and overlap from amplicon sequencing data. FEMS Microbiol Ecol [Internet]. 2020;96:fiaa131. Available from: https://doi.org/10.1093/femsec/fiaa131
